# Book Reviews

**Published:** 1912-09

**Authors:** 


### BOOK REVIEWS

An Essay on Hasheesh, Including Observations and Experiments, by *Victor Robinson* of New York, published by the Medical Review of Reviews, 80 pages, 50 cents.

This is a most interesting review of the historic and literary aspects of the drug and it contains valuable contributions to symptomatology, with a very sensible reminder that idiosyncrasy has much to do with determining the exact disturbances noted.

In passing, we may say that we have never observed the confusion of time and space so eloquently described by H. C. Wood and that the sole toxic symptom (obtained in the transition from the ordinary preparations to one of real activity) noted has been a fear of death, without any definite subjective suffering or objective indication of depression of any function to warrant the fear. 25 cents.

---

Obstetric Charts in Colors. Battle & Co., St. Louis. 25 cents.

In all, 37 figures are given, classified from normal labor to the most difficult obstetric manipulations, semi-diagramatic, so as to center the attention upon the mechanics of labor and of correcting interference. Reference to these charts will refresh the memory of the practitioner and they would be of the utmost value in the instruction of midwives, nurses and medical students. A very brief, straightforward description, with formulæ, of the preparations of the firm, concludes the book.

---

Pellagra, An American Problem, by *George M. Niles, M. D.*, Atlanta; published by W. B. Saunders Co., Philadelphia, 253 pages, illustrated, $3.00.

This is an exhaustive study by one who has had large opportunity for observing cases and patience and good judgment in reviewing the work of others. No definite conclusion is reached as to the etiology, although the author commits himself definitely to the conception of the disease as an entity, with which view, we have as definitely disagreed. The practical discussion of symptoms, diagnosis, prognosis and treatment is full and excellent, though obviously, from the point of view of the reviewer, diagnosis consists only in meeting the requirements of a somewhat arbitrary definition and prognosis, prophylaxis and treatment, not limited by the idea that we have a definite disease process, are different in some respects from the same methods applied to what is regarded as a specific disease.

---

LABORATORY METHODS, with Special Reference to the Needs of the General Practitioner, by *Drs. B. G. R. and E. G. C. Williams*, with an introduction by *Dr. Victor C. Vaughan.* Published by the C. V. Mosby Co., St. Louis, 203 pages, 42 illustrations, $2.00.

This book makes no pretentions of covering the field of clinical diagnosis but it does deal not only with the various fluids of the body but with methods of cutting and staining tissues, examining for poisons, post mortem technic and the general equipment of a simple laboratory. It does well, what it sets out to do.

---

TUMORS OF THE JAWS, by *Dr. Charles L. Scudder*, Boston. Published by the W. B. Saunders Co., Philadelphia. 391 pages, 353 illustrations, cloth, $6.00, half morocco, $7.50.

Elaborate monographs on particular fields of non-visceral surgery, of which the same author's work on Fractures, recently reviewed, is another good example, show how far specialization has advanced. But even for the general surgeon, a work of this kind is more convenient for reference than the brief chapters of several more comprehensive text books and, from the mere financial standpoint, one case in prospect would amply justify the purchase and perusal of the book. Not only is there a complete discussion of general principles and a grotesquely horrible collection of extreme cases, but technic is both described and illustrated with the utmost detail.

---

A TEXT BOOK OF GYNECOLOGY, by *Dr. William Sisson Gardner*, Baltimore; published by D. Appleton & Co. 286 pages, 138 illustrations. $3.00.

This is a very concise, systematic treatise which avoids minutiæ and gives a good general idea of the subject. It is, however, sufficiently full for all but the experienced specialist seeking the perfection of details.

---

GOULD AND PYLE'S CYCLOPEDIA OF PRACTICAL MEDICINE AND SURGERY. Second edition, revised and enlarged, under the editorship of *R. J. E. Scott, M. A., B. C. L., M. D.*, New York. 2 volumes, 653 illustrations, $14.00.

The work represents a large number of collaborators, 33 more than for the first edition and 400 pages have been added. Several very recent subjects are included. This work impresses us as being of the greatest practical value. George M. Gould has the knack of systematizing information, lighting on illustrative types, and condensing facts into tables. In this work he has been ably seconded by Walter L. Pyle. R. J. E. Scott has taken up the work and extended and revised it. For economic reasons, a cyclopedia of medicine or any other science or technical art should not represent too much invested capital nor too much requirement of time in research since, in any event, it cannot reach the maximum required of an elaborate special treatise nor can it be permanent. On the other hand, it should be more elaborate than a dicitionary whose function is merely to define words and to give a general idea of what they mean—which a formal definition usually does not do. In our opinion, this cyclopedia has struck the proper medium of size, scope and expense.

---

Diseases of the Genito-Urinary Organs and the Kidney, by *Robert Holmes Greene, A. M., M. D.*, and *Harlow Brooks, M. D.*, New York; published by the W. B. Saunders Co., Philadelphia. 3d edition, revised and enlarged, 639 pages, 339 illustrations, many of them being full page plates. $5.00.

This is a standard work, complete in its scope, already possessing the confidence of the profession. The revision brings it up to date. It would be useless to single out any part of the work for special comment.

---

Duodenal Ulcer. *B. G. A. Moynihan, M. S., F. R. C. S.*, Leeds, Eng., published by W. B. Saunders Co., Philadelphia, second enlarged edition, 486 pages, illustrated, cloth, $5.00, half morocco, $6.50.

Moynihan is one of the few great authorities on this subject. Speaking with an operative experience with 168 cases, he has had the following results:

| | | |
|---|---|---|
| Died as result of operation, | 4 | 2.14% |
| Died of other causes ...... | 4 | 2.14% |
| Cured .................... | 148 | 79.14% |
| Improved ................ | 18 | 9.6 % |
| Doubtful, no better or not traced ................. | 13 | 6.9 % |

He very frankly states that he was at first led by reports of internists to regard hyperchlorhydria as definitely diagnostic but he has modified his view as actual examination has shown it to be present in only 40% of cases. There are few conditions about which such glittering and foolish generalizations have been made, as hyperchlorhydria. It is not a very common condition, statistically, and is mimicked by various other conditions. It may, apparently, be an organic disease of itself but it is also symptomatic of many causes of irritation. While the work is mainly surgical, Moynihan views the problems broadly and his book is perhaps of greatest service to the medical practitioner, in whose hands the primary diagnosis and decision as to treatment, mainly rest.

Pellagra, by *Stewart R. Roberts, S. M., M. D.*, Atlanta. Published by the C. V. Mosby Co., St. Louis. 272 pages, 90 illustrations, $2.50.

The author has presented practically all that is known on the subject, in a systematic way and apparently without bias. He does not consider any theory as definitely proved. In particular, he opposes the time-honored corn theory and that of Mizell as to non-drying oils. While he shows that the simulium theory of conveyance is not proved, he apparently leans toward the existence of a specific organism. In a series of twenty cases, free HC1 in the gastric contents was lacking in 14 and present in normal proportion in 6 (although it must be admitted that some who very easily discover hyperchlorhydria might make this diagnosis on an acidity of 30—38 degrees.) The fæces of this whole series were stated to contain nothing not found in ordinary diarrhœa and dysentery. An allusion to indican might be of interest.

There is one point in his logic as to etiology which deserves criticism: "It cannot be produced by the poisons of both corn and protozoa." The flaw in the logic is that there may be an etiologic factor in the case of any disease which is predisposing or even necessarily cooperative with a specific infecting organism. For instance, in yellow fever, the stegomyia is not the specific cause but is practically, the determining one. In tetanus the nature of the wound in determining anærobic conditions, is practically necessary to the infectivity of the bacillus mallei. In tuberculosis for practical prophylactic and therapeutic purposes, we have got back to the old nutritive and hygienic theories of disease notwithstanding the demonstration of the bacillus. As previously stated, we do not personally regard pellagra as a specific disease at all and we hold no brief for the corn theory but it is not impossible that corn or any other article of diet or some other factor may be an essential predisposition to the implantation of a specific organism.

---

Kidney Diseases, by *W. P. Herringham, M. D., F. R. C. P.*, with chapters on Renal Diseases in Pregnancy, by *Herbert Williams, M. D., F. R. C. P., London.* Oxford Press. 378 pages, 30 illustrations, $5.50.

It should be distinctly understood that this book is not one on urinary analysis, although such methods are necessarily included and there is an especially interesting chapter on albumin. It discusses broadly, kidney diseases, including anomalies, movable kidney, the relation to cardio-vascular disease, eclampsia and many other topics. We would derive more benefit from the chemic and microscopic study of the urine if more of us read this book and if we considered the kidney as something more than an excretor of material for examination.

Tuberculin Treatment by *Clive Riviere, M. D., F. R. C. P.*, and *Egbert Morland, M. B. and B. Sc.*, the former of London, the latter of Arosa, Switzerland. The Oxford Press. 277 pages, with charts, $2.00.

While admitting the existence of human and bovine varieties, the authors consider these to be essentially identical and hence that there is no priori absurdity in using bovine tuberculin for human tuberculosis, or vice versa, in fact they consider that, at present, the choice should be according to empiric results. The statements are conservative yet favor strongly the use of tuberculin, especially in closed cases. A great many technical details deserve attention yet would lead to too lengthy a review. While we would not admit that American authors on this subject have been unduly biased by personal enthusiasm, this book representing experience in two other countries, with the elimination of local socialogic and other features, is corroborative and of value to the American physician, on account of the independent viewpoint of the authors.

---

Gonococcal Infections, by *Major C. E. Pollock* and *Major L. W. Harrison* of the Royal (British) Army Medical Corps. Oxford University Press, 222 pages, unillustrated, $2.00.

While the word gonorrhœa was coined under a false impression of its nature, the word signifying a flow of sperm, and the name gonococcus is correspondingly inaccurate, both are, nevertheless, Greek and the proper adjective is *gonococcic* and not *gonococcal*. However, the meaning is clear and the book is excellent. The authors devote considerable space to the vaccine treatment, agreeing that a good stock vaccine is all right. The chapter on gonococcic septicæmia is also especially valuable.

---

Landmarks and Surface Markings of the Human Body. *L. Bathe Rawling, M. B., B. C., F. R. C. S.*, London. Published by Paul B. Hoeber, 69 E. 59th St., N. Y., 96 pages, 29 plates, $2.00.

Some of the illustrations are photographic with added markings, some colored diagrams, most are full page, a few half page in size. All are clear. The text is arranged systematically and is much more complete than corresponding portions of standard anatomies. An appendix gives the length of various passages, tubes, etc. It is difficult to explain in a brief review just how the author has woven in a mass of interesting and practical information that renders his work of value to the general practitioner and not merely to the anatomist and operator. It may not be out of place to compliment the publisher both on the high grade of the book from the mechanic side and on his skill in selecting authors who have something out of the ordinary to present to readers.

